# A Novel Strategy for Introducing Metal-Organic Frameworks into Carbon Fiber to Improve the Interfacial and Mechanical Properties of Carbon Fiber/Epoxy Composites

**DOI:** 10.3390/ma18214856

**Published:** 2025-10-23

**Authors:** Jin Yan, Hongyi Ma, Qiyu Deng, Hongyun Li, Lei Xiong

**Affiliations:** 1 Composite Testing Technology Center, AVIC Composite Co., Ltd., Beijing 101300, China; 2 School of Materials Science and Engineering, Nanchang Hangkong University, Nanchang 330063, China

**Keywords:** carbon fiber, ZIF90 nanocrystals, interfacial adhesion, mechanical properties, composites

## Abstract

The interfacial properties in carbon fiber (CF)-reinforced polymer composites are substantially limited by the chemically inactive and smooth CF surfaces. In this study, zeolitic imidazolate framework 90 (ZIF90) was chemically grafted onto CF surfaces via polyethyleneimine (PEI) as a coupling agent to construct a hierarchical reinforcement interface in CF/epoxy composite. The successful synthesis of CF grafted with PEI and ZIF90 (CF-PEI-ZIF90) was systematically characterized by Fourier-transform infrared spectroscopy (FTIR), X-ray photoelectron spectroscopy (XPS), thermogravimetric analysis (TGA), scanning electron microscopy (SEM), and X-ray diffraction (XRD). The incorporation of ZIF90 nanocrystals and PEI molecules into CF surfaces effectively improved interfacial adhesion through mechanical interlocking and chemical interactions, thereby optimizing stress transfer efficiency at the fiber–matrix interface and improving the interfacial properties of the composite. Additionally, the resultant CF-PEI-ZIF90/epoxy composite demonstrated significant mechanical enhancement, with the tensile and bending strengths increasing by 33.5% and 21.4%, respectively, compared to unmodified CF/epoxy composites. This work provides a novel strategy for enhancing the interfacial performance of CF composites by leveraging the unique properties of metal-organic frameworks, which is critical for advancing high-performance structural materials in aerospace and automotive applications.

## 1. Introduction

Carbon fiber-reinforced polymer (CFRP) composites are increasingly recognized as high-performance structural materials due to their outstanding specific strength, excellent damage tolerance, and superior fatigue characteristics. These attributes have led to their widespread adoption in advanced engineering applications, particularly in aerospace engineering, defense systems, and automotive light weighting initiatives [1,2,3]. However, the full exploitation of these composites’ mechanical potential is significantly constrained by the weak interfacial adhesion between CF and the polymer matrix [4,5]. This limitation primarily is caused by the inherently low surface energy and chemical inertness of CF surfaces, which results in poor fiber–matrix interactions. Moreover, the substantial modulus mismatch between the high-stiffness CF and relatively compliant thermoset matrix creates stress concentration phenomena at the interface under external load [6,7,8]. These localized stress concentrations frequently initiate interfacial debonding and subsequent crack propagation, ultimately leading to premature composite failure. Consequently, the strategic engineering of interfacial properties in CFRP composites has emerged as a critical research focus for optimizing their mechanical performance.

Current research has demonstrated that the surface functionalization of CF with nanomaterials, including nanoparticles and two-dimensional nanosheets, can significantly improve interfacial adhesion and stress transfer efficiency in composite materials [9,10,11]. While these nanoscale modifications show promising results, several technical challenges remain that limit their practical implementation. The randomness of chemical grafting reactions makes precise control over nanoparticle distribution difficult to achieve, often resulting in non-uniform surface coverage. Additionally, the dense packing of nanoparticles can impede resin infiltration during composite fabrication, creating localized regions of poor matrix penetration [12,13,14,15]. Recent efforts have focused on optimizing reaction conditions and exploring alternative functionalization strategies to address these challenges while maintaining the performance benefits of nanoscale surface modifications.

Metal-organic frameworks (MOFs) represent a promising class of crystalline porous materials that combine inorganic metal clusters with organic linkers, offering unique advantages including tunable pore structures, exceptional surface areas, and versatile chemical functionality [16,17,18,19]. As one of the most important primary members of MOFs, ZIF90 is constructed from Zn^2+^ tetrahedra via a bridge of imidazole-2-carboxaldehyde and can be produced through a simple preparation process. Compared with other popular MOFs, ZIF90 is characterized for its robust zeolite-like architectures, which combine excellent thermal and chemical stability with precisely defined micropores. In addition, ZIF90 is another analog of ZIF-8 and ZIF-67 with a large amount of aldehyde groups on the imidazole ligands (imidazole-2-carboxaldehyde), which allows for increasing the active groups of the CF surface [20,21]. Therefore, the surface modification of CF surfaces with ZIF90 nanocrystals offers a promising approach for enhancing composite interfacial properties. The incorporation of ZIF90 creates a nanoscale surface topography that significantly increases fiber surface roughness and effective contact area, thereby promoting mechanical interlocking at the fiber–matrix interface. Additionally, the abundant nitrogen-containing functional groups in ZIF90 improve surface polarity for CF and provide active sites for chemical interactions with polymer matrices. The porous architecture of ZIF90 further facilitates resin impregnation, potentially forming an interpenetrating network that enhances matrix toughening [22,23]. Despite these advantages, practical implementation faces challenges common to nanoparticle-based modifications, particularly in achieving high grafting density and uniform dispersion of ZIF90 crystals across fiber surfaces. These limitations must be addressed to fully realize the potential of this surface engineering strategy for composite applications.

In this study, we present a novel approach for fabricating CF-PEI-ZIF90 hybrid reinforcements through the in situ growth of ZIF90 nanocrystals, utilizing PEI as a coupling agent to covalently anchor ZIF90 onto the CF surface, to improve the interfacial and mechanical properties for composite materials. The incorporation of PEI molecules can improve grafting density and uniformity for ZIF90, and also increase active groups on CF surfaces. Comprehensive characterization including FTIR, XPS, XRD, SEM, TGA, and dynamic contact angle measurements was conducted to evaluate the chemical composition, morphological changes, and surface wettability of modified fibers. Furthermore, epoxy matrix composites reinforced with CF-PEI-ZIF90 hybrid fibers were fabricated and mechanically tested to systematically evaluate the effects of this surface modification.

## 2. Materials and Methods

### 2.1. Materials

PAN-based CF (T300, density 1.75 g/cm^3^, diameter 7~8 μm) was provided by Toray Corporation (Tokyo, Japan), and the sizing agent had been completely removed, which was recorded as unmodified CF. Zinc nitrate hexahydrate (98% purity) was produced in Damao Chemical Reagent Factor (Tianjin, China). Polyethyleneimine (PEI, 99% purity) was from Sahn Chemical Technology Co., Ltd (Shanghai, China). The resin (Epoxy 51, 97% purity) and its hardener (methyltetrahy-drophthalic anhydride, 98% purity) were received from Fengguang Chemicals Corp (Yingkou, China). γ-Glycidoxypropyltrimethoxysilane (KH560, 98% purity) and imidazole-2-carboxaldehyde (98% purity) were purchased from Anhui Zeseng Tech Corp (Hefei, China). All other chemicals were purchased from Aldrich Corp (Milwaukee, WI, USA).

### 2.2. Preparation of Epoxy Group-Functionalized CF

The unmodified CFs were subjected to oxidative treatment by refluxing in concentrated nitric acid (68 wt.%) at 80 °C for 6 h to introduce oxygen-containing functional groups. After reaction, the fibers were thoroughly rinsed with deionized water until pH reached neutrality, ensuring the complete removal of residual acid. The oxidized CF (denoted as CF-OH) were then dried in a vacuum oven at 60 °C for 12 h. For silane functionalization, a homogeneous reaction solution was prepared by mixing anhydrous ethanol, deionized water, and γ-glycidoxypropyltrimethoxysilane (KH560) in a volume ratio of 90:5:5. The solution was transferred into a round-bottom flask, and CF-OH was immersed in the mixture. The dispersion was sonicated for 30 min, followed by the addition of dilute hydrochloric acid to adjust the pH to 3–4. The system was then refluxed at 80 °C for 12 h to facilitate covalently introducing the silane coupling agent into the fiber surface. Finally, the modified fibers were washed repeatedly with ethanol to remove unreacted silane and dried at 60 °C under vacuum to obtain epoxy group-functionalized CF (CF-KH560).

### 2.3. Preparation of CF-PEI-ZIF90

**Scheme 1:**
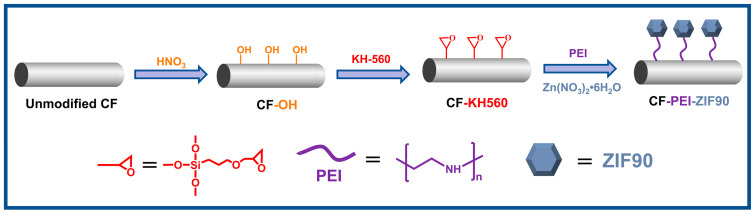
Schematic representation for surface modification of CF with PEI and ZIF90.

Firstly, CF-KH560 and polyethyleneimine (PEI) were dispersed in methanol and subjected to reflux conditions at 60 °C for 5 h, followed by gradual cooling to ambient temperature. Subsequently, stoichiometric amounts of zinc nitrate hexahydrate and imidazole-2-carboxaldehyde were sequentially introduced into the reaction mixture under continuous stirring until complete dissolution was achieved. The reaction was allowed to proceed for 4 h to ensure complete ZIF90 formation on fiber surfaces. The modified fibers were then purified through multiple methanol washing cycles until the supernatant exhibited optical clarity, indicating the removal of unreacted precursors and byproducts. Final drying at 60 °C for 48 h yielded CF-PEI-ZIF90 hybrid reinforcements. A schematic representation of the complete surface modification process is provided in Scheme 1.

### 2.4. Preparation of Composites

A microdroplet test was employed to effectively assess the interfacial performance of epoxy composites. The microdroplets, consisting of epoxy 51 and methyltet-rahydrophthalic anhydride in a mass ratio of 100:80, were applied to the monofilament and subsequently cured at 110 °C for 2.5 h and 165 °C for another 1 h. The composites used for mechanical testing were fabricated using a hot-molding method, ensuring complete fiber infiltration in a mixture of epoxy and hardener, while maintaining a fiber volume fraction of 60 ± 2%. After preheating the mold to 90 °C for approximately 30 min, the infiltrated fibers were placed within it. The curing process for the composites involved applying 2.5 h at 110 °C, 2 h at 140 °C, and 1 h at 165 °C.

### 2.5. Characterization

The surface functional groups of fibers were analyzed by Fourier-transform infrared spectroscopy (FTIR, Shimadzu IR Prestige-21, Shimadzu, Kyoto, Japan) in the range of 4000–5000 cm^−1^. Morphological characterization was performed on a field-emission scanning electron microscope (FE-SEM, Nova NanoSEM 450, Thermo Fisher Scientific, Waltham, MA, USA) with a lens detector (TLD) technique and at an accelerating voltage of 15 kV, with samples sputter-coated with gold prior to imaging to enhance conductivity. The surface chemical composition was determined by X-ray photoelectron spectroscopy (XPS, Thermo Scientific ESCALAB 250Xi, Thermo Fisher Scientific, Waltham, MA, USA) using monochromatic Al Kα radiation. Contact angle measurements were conducted using a sessile drop method with water and diiodomethane as detected liquids. The thermal stability was evaluated by thermogravimetric analysis (TGA, TA Instrument, New Castle, DE, USA Q50) under a nitrogen atmosphere with a heating rate of 10 °C/min from ambient temperature to 800 °C. The crystalline structure was examined by X-ray diffraction (XRD, Bruker, Billerica, MA, USA, D8 Advance) using Cu Kα radiation with a scanning range of 5–50° (2θ). The mechanical properties were assessed using a universal testing machine (WDW-100, Jinan Chengyu Testing Equipment, Jinan, China) according to GB/T1447-2005 (tensile strength) and GB/T1449-2005 (bending strength). The interfacial shear strength (IFSS) between fiber and resin was evaluated using a micro-droplet debonding test.

## 3. Results and Discussion

### 3.1. Physicochemical Characterizations for Modified CFs

**Figure 1:**
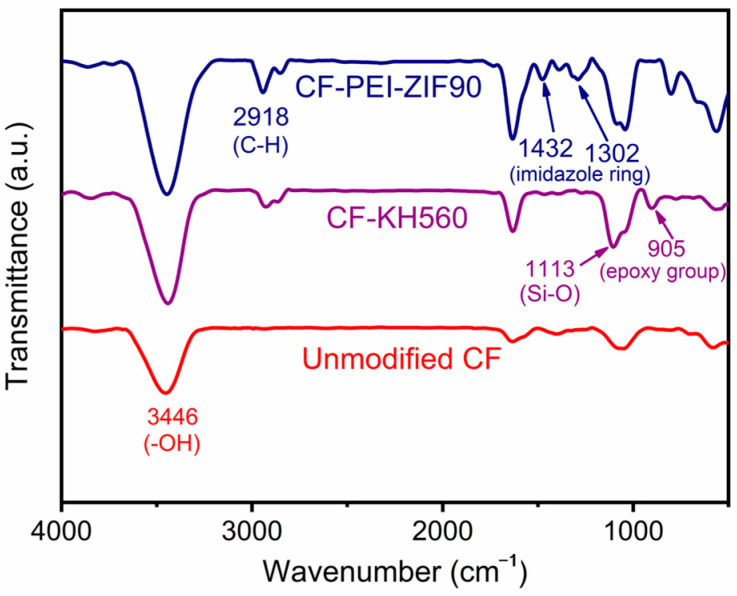
FTIR spectra of unmodified CF, CF-KH560, and CF-PEI-ZIF90.

FTIR analysis was performed to characterize the chemical modifications of CF at different stages, as depicted in Figure 1. The spectrum of unmodified CF shows no distinctive functional group peaks in the fingerprint region (except for the peak at 3446 cm^−1^ corresponding to -OH groups), indicating the inert nature of unmodified CF. After silane treatment (CF-KH560), two new absorption bands emerged at 1113 cm^−1^ and 905 cm^−1^, corresponding to the Si-O stretching vibration and epoxy group vibration, respectively, confirming the successful grafting of the KH560 coupling agent. Further modification with polyethyleneimine and ZIF90 (CF-PEI-ZIF90) introduced two additional characteristic peaks at 1432 cm^−1^ (imidazole ring stretching) and 1302 cm^−1^ (imidazole ring in-plane bending). These vibrational signatures provide direct evidence for the incorporation of ZIF90 nanocrystals into the CF surface. Additionally, the FTIR spectrum of CF-PEI-ZIF90 exhibits a significant enhancement in C-H vibration (2918 cm^−1^) compared to CF-KH560. This notable intensity increase can be attributed to the introduction of polyethyleneimine molecules during the surface modification process. The FTIR analysis collectively demonstrates the functionalization of CF, with each modification stage producing distinct spectral features corresponding to the introduced functional groups.

**Figure 2:**
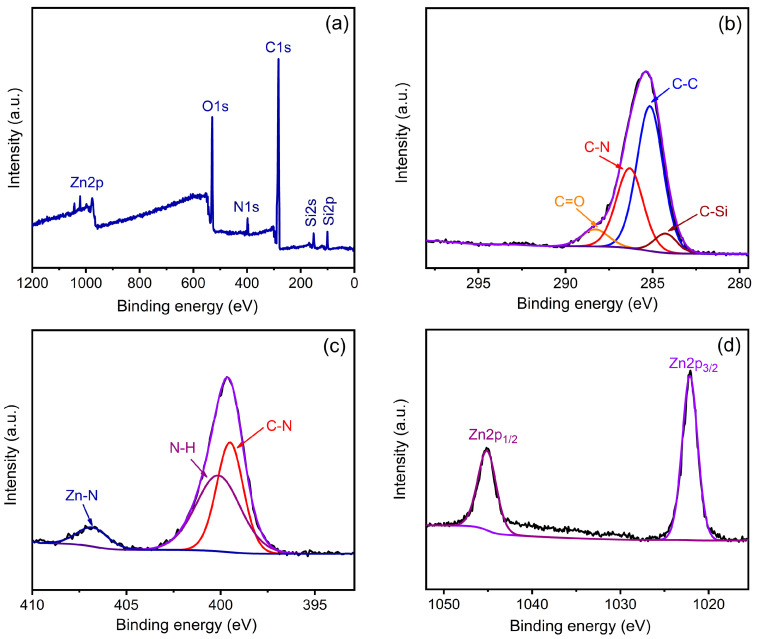
XPS wide-scanning spectrum of CF-ZIF90 (**a**) and peak-fitting curves for C1s peak (**b**), N1s peak (**c**), and Zn2p peak (**d**).

X-ray photoelectron spectroscopy (XPS) analysis was employed to investigate the surface chemical composition of CF-PEI-ZIF90. The survey spectrum (Figure 2a) reveals five distinct peaks at binding energies of 285.7 (C1s), 532.3 (O1s), 400.5 (N1s), 101.8 (Si2p), and 1022.8 eV (Zn2p), confirming the presence of these elements in modified CF. High-resolution spectra were subsequently analyzed to identify specific chemical states. The C1s spectrum (Figure 2b) could be deconvoluted into four components: 284.3 eV (C-Si, from the KH560 coupling agent), 285.2 eV (C-C, graphitic carbon), 286.4 eV (C-N, attributed to both PEI and imidazole-2-carboxaldehyde), and 288.2 eV (C = O, carboxyl groups). The N1s spectrum (Figure 2c) exhibits three characteristic peaks at 399.4 eV (imine groups from PEI), 400.2 eV (C-N bonds), and 406.9 eV (Zn-N coordination), the latter indicating successful metal–ligand coordination between zinc ions and nitrogen-containing functional groups. Furthermore, the Zn2p region shows two spin–orbit components at 1022.1 eV (Zn2p3/2) and 1045.2 eV (Zn2p1/2), with the characteristic 23.1 eV splitting and binding energies consistent with the formation of ZIF90 nanocrystals on fiber surfaces. These XPS results collectively demonstrate the successful grafting of ZIF90 and PEI molecules onto CF.

**Figure 3:**
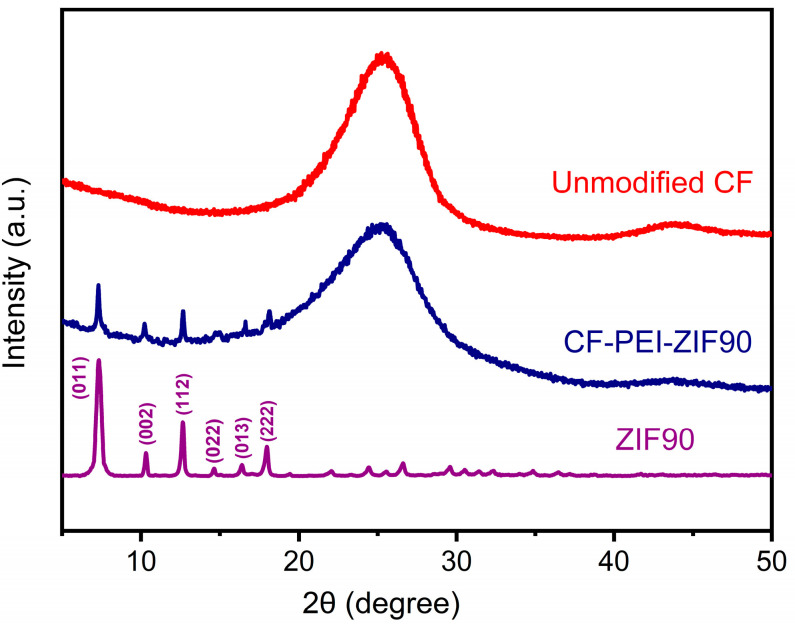
XRD patterns for ZIF90, unmodified CF, and CF-PEI-ZIF90.

X-ray diffraction (XRD) patterns of ZIF90, unmodified CF, and CF-PEI-ZIF90 are presented in Figure 3. The characteristic (002) diffraction peak of graphitic carbon at 2θ = 25.4° is observed in both unmodified CF and CF-PEI-ZIF90 samples, confirming that the fundamental carbon structure remains intact after modification. However, the reduced peak intensity in CF-PEI-ZIF90 suggests partial surface disordering induced by the grafting process. The pure ZIF90 pattern exhibits characteristic diffraction peaks at 2θ = 7.3°, 10.3°, 12.7°, and 18.0°, corresponding to the (011), (002), (112), and (222) crystal planes, respectively, consistent with the XRD pattern of crystalline ZIF90 reported in the literature [24]. Importantly, these distinctive ZIF90 diffraction features are clearly discernible in the CF-PEI-ZIF90 pattern, providing definitive evidence for successful ZIF90 immobilization on CF surfaces.

**Figure 4:**
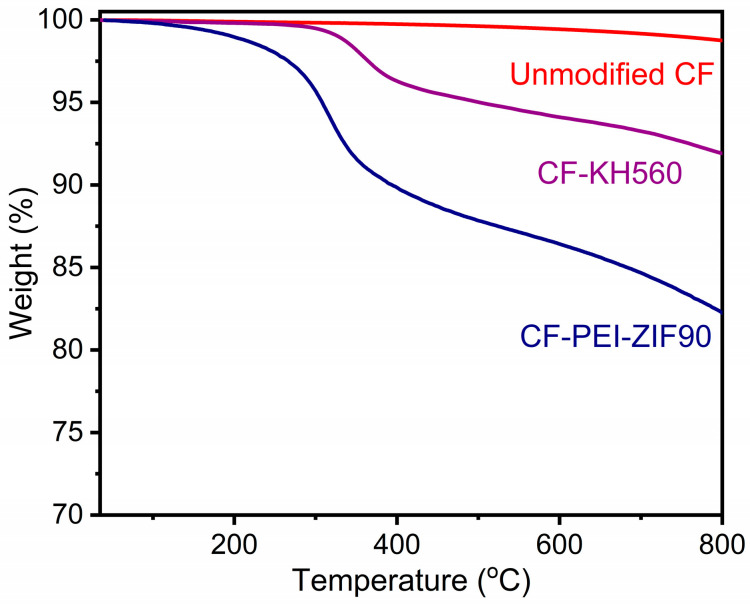
TGA curves of unmodified CF, CF-KH560, and CF-PEI-ZIF90.

Thermogravimetric analysis (TGA) was conducted to assess the thermal stability evolution of CFs throughout the modification process. As illustrated in Figure 4, the unmodified CF displays exceptional thermal stability, with merely 1.3% mass loss between 35 and 800 °C, attributable to moisture evaporation and the minor decomposition of surface oxygen functionalities. CF-KH560 demonstrates a more pronounced weight loss of 8.1% over the same temperature range, corresponding to the thermal degradation of the grafted KH560 coupling agent. Notably, CF-PEI-ZIF90 exhibits the most significant thermal decomposition behavior, with a total mass loss of 17.7%. A marked weight reduction occurs at approximately 340 °C, reflecting the combined decomposition of both polyethyleneimine and ZIF90 nanocrystals. These progressive changes in thermal decomposition profiles provide compelling evidence for the successful sequential functionalization of CF. The increasing weight loss percentages at each modification stage directly correlate with the cumulative organic and inorganic components introduced through surface modification.

### 3.2. Surface Wettability and Morphology of CFs

**Figure 5:**
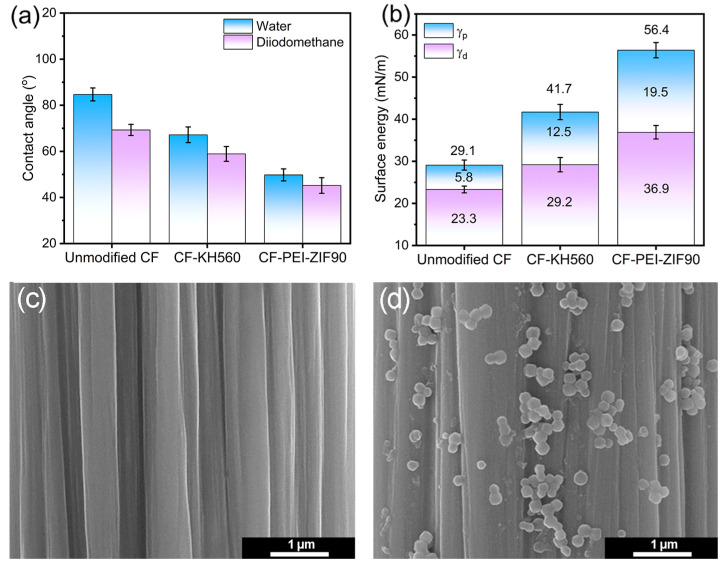
CAs (**a**) and surface energies (**b**) of CFs; surface morphologies of unmodified CF (**c**) and CF-PEI-ZIF90 (**d**).

The interfacial performance of carbon fiber-reinforced composites is fundamentally determined by the surface energy characteristics of reinforcing fibers, which directly determine their wettability with the polymer matrix. To quantitatively evaluate these critical surface properties, dynamic contact angle measurements were performed on variously modified CFs, with subsequent calculation of surface energy components. Figure 5a,b present the contact angles (CAs) and derived surface energies for unmodified CF, CF-KH560, and CF-PEI-ZIF90. The unmodified CF exhibits water and diiodomethane contact angles of 84.7° and 69.3°, respectively, corresponding to a total surface energy of 29.1 mN/m. Silane treatment with KH560 yields measurable improvements, reducing the contact angles to 67.3° (water) and 58.9° (diiodomethane) while increasing the surface energy to 41.7 mN/m, representing a 43.3% enhancement attributable to the introduced polar functional groups. The most substantial modification occurs with CF-PEI-ZIF90, which demonstrates significantly reduced contact angles of 49.8° (water) and 45.2° (diiodomethane). The corresponding surface energy reaches 56.4 mN/m, representing a remarkable 93.8% increase compared to unmodified CF. This dramatic improvement is due to two complementary mechanisms: the nanoscale surface roughness induced by ZIF90 growth enhanced the dispersive component (γd) of surface energy, while the abundant active functional groups from both ZIF90 and PEI contributed to increased polar component (γp). These surface energy modifications have profound implications for composite performance. The substantially improved wettability promotes more contact sites between fiber and resin during composite fabrication, while the increased surface energy provides stronger thermodynamic driving force for adhesion. The combination of enhanced dispersive and polar components facilitates both physical and chemical bonding across the interface, ultimately leading to improved stress transfer efficiency in the resulting composites. This comprehensive surface characterization provides fundamental understanding of how molecular-scale modifications translate to macroscopic interfacial property improvements.

Surface morphological characterization of unmodified and modified CF was performed using SEM to evaluate the effects of stepwise functionalization process. The SEM images in Figure 5 reveal distinct differences between unmodified CF and modified CF surfaces. The unmodified CF (Figure 5c) displays a characteristically smooth surface morphology with shallow longitudinal grooves running parallel to the fiber axis. This relatively featureless topography, while typical of commercial carbon fibers, provides limited mechanical interlocking sites at the fiber–matrix interface, potentially compromising composite interfacial strength. In contrast, CF-PEI-ZIF90 (Figure 5d) exhibits a completely transformed surface architecture. High-magnification images clearly show uniform coverage of polyhedral nanoparticles, measuring approximately 50–100 nm in diameter, distributed across the entire fiber surface. These nanoparticles display the characteristic rhombic dodecahedral morphology of ZIF90 crystals, consistent with previous reports in the literature [25]. The successful growth of ZIF90 creates a nanoscale textured surface that significantly enhances both surface area and mechanical interlocking potential.

### 3.3. Interfacial Properties of Composites

**Figure 6:**
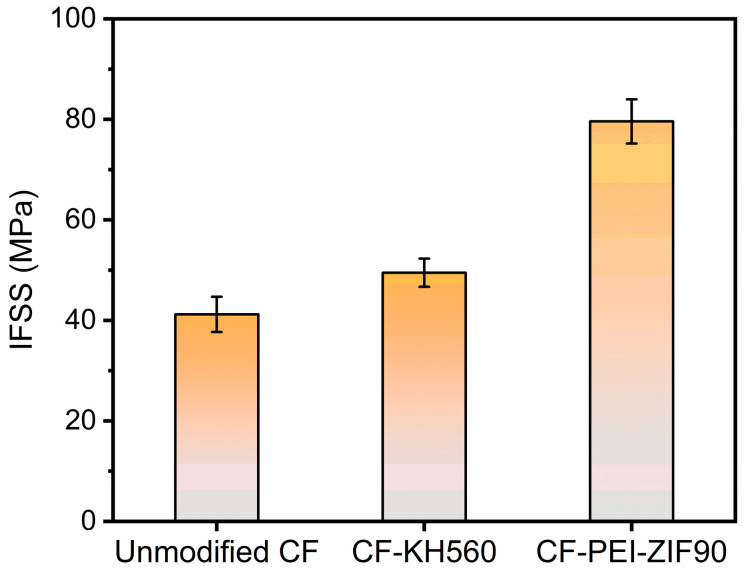
IFSS of unmodified CF, CF-KH560, and CF-PEI-ZIF90.

The interfacial shear strength (IFSS) between carbon fibers and the epoxy matrix serves as a key indicator of bonding quality at the fiber–matrix interface, directly reflecting load transfer efficiency in composite materials. As illustrated in Figure 6, the baseline IFSS measurement for the unmodified CF composite registers at 41.2 MPa, a relatively low value consistent with the smooth, chemically inert nature of CF surfaces. Initial surface modification through nitric acid oxidation followed by KH560 treatment yields moderate improvement, elevating IFSS to 49.5 MPa through the introduction of reactive silane functional groups. The most substantial enhancement is achieved with CF-PEI-ZIF90, which demonstrates a remarkable IFSS value of 79.6 MPa, representing a 93.2% increase over unmodified CF. The achieved 93.2% improvement in IFSS significantly surpasses the typical gains of 39.4–40.5% reported in the literature for other MOF-based modification strategies [26,27].

This dramatic improvement originates from multiple synergistic mechanisms operating at the fiber–matrix interface. The ZIF90 nanocrystals create nanoscale surface topography that simultaneously increases effective contact area and promotes mechanical interlocking through their distinctive crystalline morphology. Meanwhile, the abundant N-H groups presented in both ZIF90 and PEI participate in covalent bonding with epoxy groups during the curing process, establishing robust chemical linkages across the interface. Additional contributing factors include the improved surface wettability resulting from introduced polar groups and the unique stress distribution characteristics enabled by ZIF90’s porous architecture. The combination of these effects creates an optimized interface that effectively transfers shear stresses while resisting debonding, as evidenced by the significantly enhanced IFSS performance. These interfacial modifications collectively address the fundamental limitations of carbon fiber–epoxy systems by transforming the traditionally weak physical interface into a strongly bonded interphase region [21,28].

**Figure 7:**
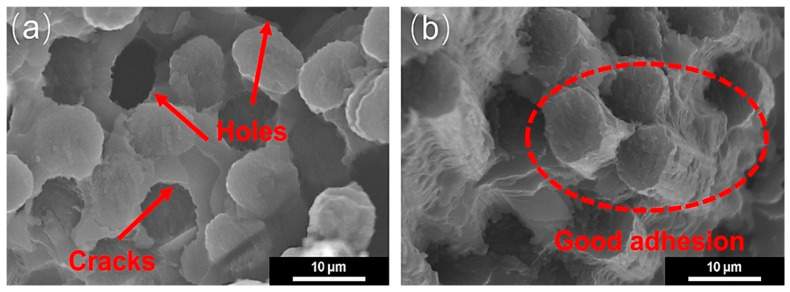
Fracture surfaces of unmodified CF/epoxy composite (**a**) and CF-PEI-ZIF90/epoxy composite (**b**).

The interfacial bonding characteristics of CF/epoxy composites were investigated through fracture surface analysis using SEM. When composite materials fracture under external stress, the resulting morphology provides critical information about interface quality and failure mechanisms, serving as an effective means to evaluate the success of chemical modification strategies. Figure 7a presents the fracture morphology of the unmodified CF/epoxy composite, revealing several characteristic features indicative of poor interfacial adhesion. Numerous voids and cracks are evident in Figure 7a, and it also displays clean fiber surfaces completely detached from the epoxy matrix. These observations demonstrate the consequences of insufficient interfacial bonding under applied stress; failure preferentially occurs at the weak fiber–matrix interface through debonding mechanisms before fiber fracture can occur. This phenomenon is caused by the lack of active functional groups on unmodified CF surfaces, which limits interfacial interactions with the epoxy matrix.

In contrast, the fracture surfaces of the CF-PEI-ZIF90/epoxy composite (Figure 7b) exhibit markedly different morphology. The absence of voids and cracks, coupled with substantial resin residue adhering to pulled-out fibers, provides clear evidence of enhanced interfacial bonding. This improved morphology results from the successful surface modification, which introduced active functional groups and nanoscale surface features that promote both chemical and mechanical interlocking. The fracture pattern indicates a shift in failure mechanism; load transfer occurs efficiently across the strengthened interface, leading to cohesive failure within either the matrix or fiber rather than interfacial debonding. This comparative analysis demonstrates that the chemical grafting modification effectively transforms the failure mode from adhesive failure to coexisting interfacial adhesion failure and cohesive failure, confirming substantial improvements in load transfer efficiency and interfacial strength. The fracture surface characteristics correlate well with the measured interfacial property enhancements, providing microstructural evidence for the success of the surface modification approach.

### 3.4. Mechanical Properties of Composites

**Figure 8:**
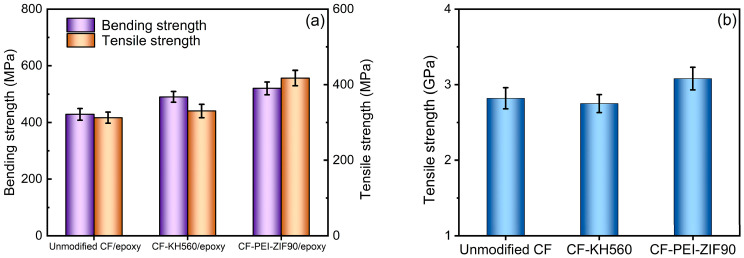
Bending strength and tensile strength of various composite systems (**a**) and Monofilament tensile strength of surface-modified carbon fibers (**b**).

The mechanical properties of composites with different surface treatments were systematically evaluated through three-point bending and tensile strength tests. As shown in Figure 8a, the unmodified CF/epoxy composite exhibits mechanical properties with a bending strength of 428.6 MPa and a tensile strength of 312.7 MPa. Surface modification with the KH560 silane coupling agent results in moderate improvements, while the most significant enhancement is achieved with the CF-PEI-ZIF90/epoxy composite, demonstrating superior bending strength (520.4 MPa) and tensile strength (417.5 MPa). These values represent substantial increases of 21.4% and 33.5%, respectively, compared to the unmodified CF/epoxy composite.

The remarkable mechanical improvement can be attributed to synergistic effects from the grafting PEI and ZIF90. Progressive chemical modification introduces increasing amounts of reactive groups (-NH2, -OH, and epoxy) that enhances fiber surface wettability and facilitates covalent bonding with the epoxy matrix. Furthermore, the incorporated ZIF90 nanocrystals create nanoscale surface roughness while providing additional organic ligands for chemical interaction. This dual-phase interfacial enhancement, combining chemical bonding and mechanical interlocking, effectively improves stress transfer efficiency across the interface [23,27,28]. The modified interface demonstrates reduced stress concentrations and suppressed premature debonding, leading to more efficient utilization of carbon fiber’s intrinsic mechanical properties. The mechanical test results correlate well with microscopic observations of fracture surfaces, which shows a transition from adhesive failure in unmodified composites to cohesive failure in the modified system. This shift in failure mode confirms that the surface treatment successfully strengthened the typically weak interface while maintaining the structural integrity of both reinforcement and matrix phases.

The monofilament tensile test was further utilized to evaluate the influence of surface modifications on fiber strength. As shown in Figure 8b, the tensile strength of unmodified CF measures 3.11 GPa. After acid and KH560 treatment, the strength decreases to 2.97 GPa, corresponding to a reduction of 4.5%. Although acid oxidation effectively introduces oxygen-containing functional groups such as hydroxyl groups into the fiber surface, the etching effect of nitric acid also induces surface defects, leading to a noticeable degradation of tensile strength. In contrast, the CF-PEI-ZIF90 sample exhibits a significant increase in tensile strength, reaching 3.38 GPa, an increase of 8.7% relative to the unmodified CF. This enhancement can be attributed to two main factors: (1) ZIF90 nanoparticles and PEI filled surface micro-defects and voids, thereby alleviating stress concentration; and (2) the shear interactions between ZIF90 and the fiber surface contributed to distributed load dissipation.

## 4. Conclusions

A novel surface modification strategy was developed to in situ grow ZIF90 nanocrystals on carbon fibers using polyethyleneimine as a molecular bridge. This modification strategy resulted in substantial changes to CF surface characteristics, including a marked increase in active functional groups derived from both PEI molecules and ZIF90 frameworks. Meanwhile, the incorporation of ZIF90 nanocrystals created a nanoscale textured surface morphology, significantly enhancing the surface roughness and effective contact area available for matrix interaction. These surface alterations led to improved interfacial adhesion in the resulting composites through synergistic mechanisms. The introduced functional groups facilitated chemical bonding with the epoxy matrix, while the nanoscale surface topography promoted mechanical interlocking. This dual-phase interfacial enhancement enabled more efficient stress transfer during mechanical loading, effectively mitigating stress concentration phenomena at the fiber–matrix interface, and finally significantly improving the interfacial properties of the composite. Quantitative mechanical testing revealed substantial property improvements, with the modified CF-PEI-ZIF90/epoxy composite demonstrating an IFSS of 79.6 MPa, a bending strength of 520.4 MPa, and a tensile strength of 417.5 MPa, representing increases of 93.2%, 21.4%, and 33.5%, respectively, compared to the unmodified CF/epoxy composite. This work demonstrates a practical and effective approach for fabricating high-performance structural composites with outstanding interfacial and mechanical properties.

## Data Availability

The original contributions presented in this study are included in the article. Further inquiries can be directed to the corresponding authors.
